# No influence of posterior tibial slope change on outcomes after cruciate-retaining total knee arthroplasty: a prospective cohort study

**DOI:** 10.1007/s00402-022-04653-5

**Published:** 2022-10-15

**Authors:** Francisco A. Miralles-Muñoz, Emilio Sebastia-Forcada, Adolfo Perez-Aznar, Matias Ruiz-Lozano, Blanca Gonzalez-Navarro, Alejandro Lizaur-Utrilla

**Affiliations:** 1 Department of Orthopedic Surgery, Elda University Hospital, Ctra Elda-Sax s/n, Elda, 03600 Alicante, Spain; 2 Department of Traumatology and Orthopedics, Miguel Hernandez University, Avda Universidad s/n, San Juan de Alicante, 03202 Alicante, Spain

**Keywords:** Cruciate retaining, Total knee arthroplasty, Posterior tibial slope, Range of motion, Functional outcome

## Abstract

**Objective:**

To investigate whether the functional outcomes were affected by the change in posterior tibial slope (PTS) after using a predetermined PTS for primary cruciate-retaining total knee arthroplasty (CR-TKA).

**Methods:**

Prospective cohort study of 152 patients who underwent primary CR-TKA with a standardized PTS of 5º regardless of the native PTS. Patients were classified postoperatively in two ways. Firstly, according to the PTS change from preoperative to postoperative (increased or decreased PTS group). Secondly, according to the PTS difference between preoperative and postoperative ≤ 4º (group A) and > 4º (group B). The functional outcomes were assessed with the Knee Society Scores (KSS), McMaster Universities Osteoarthritis Index questionnaire (WOMAC), and range of motion (ROM). Preoperative and postoperative PTS were measured on lateral knee radiographs.

**Results:**

The minimum follow-up was 5 year. There were no significant differences at the final follow-up in functional outcomes between increased (88 patients) and decreased (64 patients) PTS groups. Likewise, there were no significant differences in functional outcomes between group A (79 patients) and group B (73 patients). In multivariate analysis, the PTS change was not significant predictor for improvement in functional outcome (OR 1.08; 95% CI 0.70–1.40; *p* = 0.061).

**Conclusion:**

The PTS change between preoperative and postoperative has no influence on the functional outcomes using a CR-TKA. A standardized PTS regardless of the native is a reliable procedure for primary CR-TKA.

## Introduction

Total knee arthroplasty (TKA) is an effective procedure in relieving pain and restoring function for most patients with end-stage degenerative knee [1]. The influence of the posterior tibial slope (PTS) on the range of motion (ROM) after cruciate-retaining TKA (CR-TKA) [2, 3] or posterior-stabilized TKA (PS-TKA) [4, 5] has been extensively discussed. However, most studies focused their results only on postoperative ROM but not reporting other functional outcomes. Only a few reported functional data associated to postoperative PTS change after CR-TKA [3, 6] or PS-TKA [4, 7], but only two of them were prospective studies [4, 6] and their follow-up was less than one year, and only one using CR-TKA [6]. Thus, the evidence for the effect of the postoperative PTS change on CR-TKA functional outcomes at least the medium term is very limited. On the other hand, while some authors advise reproducing the native PTS to optimize postoperative function [3, 8], others recommend increasing the PTS [9, 10], and still others use a default PTS for all patients [2, 4].

The objective of this study was to investigate whether the PTS change after CR-TKA affected other functional outcomes than ROM. The hypothesis was that the PTS change from preoperative to postoperative had no influence on the functional outcomes.

## Materials and methods

This cohort prospective comparative study was approved by the institutional ethics committee requiring informed consent of the patients. Between February 2015 and June 2016, consecutive patients who underwent primary CR-TKA were invited to participate. The inclusion criterion was primary osteoarthritis. CR-TKA indication was a competent posterior cruciate ligament assessed preoperatively and intraoperatively. A minimum follow-up of 5 years was required for result analysis. The exclusion criteria were PS-TKA, inflammatory or posttraumatic arthritis, prior knee surgeries including osteotomy or anterior cruciate ligament reconstruction, severe preoperative knee contracture (flexion less than 60º or extension lag more than 20º), and varus/valgus deformity more than 20º. To minimize confounding variables, patients requiring TKA revision were also excluded.

Firstly, a comparative analysis was performed between patients with increased postoperative PTS compared to preoperative and those with decreased postoperative PTS. Considering that a PTS difference of 4º between preoperative and postoperative did not influence functional outcome [7], a second comparative analysis was performed between patients with a difference ≤ 4° (group A) and those with a difference > 4º (group B).

### Surgical protocol

All surgeries were performed or supervised by the same surgeon. A standard operative technique was used in all patients with an anterior midline skin incision and medial parapatellar approach. Standard TKA instruments with femoral and tibial intramedullary cutting guides from the Trekking knee modular system (Samo Biomedica, Bologna, Italy) were used. All implants were a cruciate-retaining design with hybrid fixation. Patella was resurfaced in all patients. The tibial cut was perpendicular to the tibia axis in the coronal plane, and a 5º tibial cutting block was used in all patients regardless of their native PTS. The distal femoral cut was performed using a cutting block between 4 and 6º valgus. Flexion and extension gaps were checked.

All patients received standardized antibiotic and thromboembolic prophylaxis. Active knee motion under the supervision of a physiotherapist was started on the second postoperative day, and immediate weight-bearing with a walker was allowed.

### Evaluation and outcome variables

Clinical and radiological evaluations were made preoperatively and postoperatively at 3 and 6 months, 1 year, and then biannually until at least 5 years. The primary outcome was the Knee Society Scores (KSS) [11]. For KSS scores, minimal clinically important difference (MCID) was 10, and a substantial clinical benefit (SCB) was 40 [12]. MCID represents the minimal change between the preoperative and postoperative in a clinical score that the patient perceives to be beneficial, while SCB is the postoperative improvement that the patient perceives as clinically relevant. The McMaster Universities Osteoarthritis Index questionnaire (WOMAC) [13] was also used to assess quality of life. The WOMAC was transformed to a 0–100 scale, so a higher score implied a better outcome. ROM was measured in the supine position using a clinical goniometer. Clinical assessments were performed by two independent surgeons, and the interobserver reliability for KSS scores had an intraclass correlation coefficient (ICC) of 0.814.

**Fig. 1:**
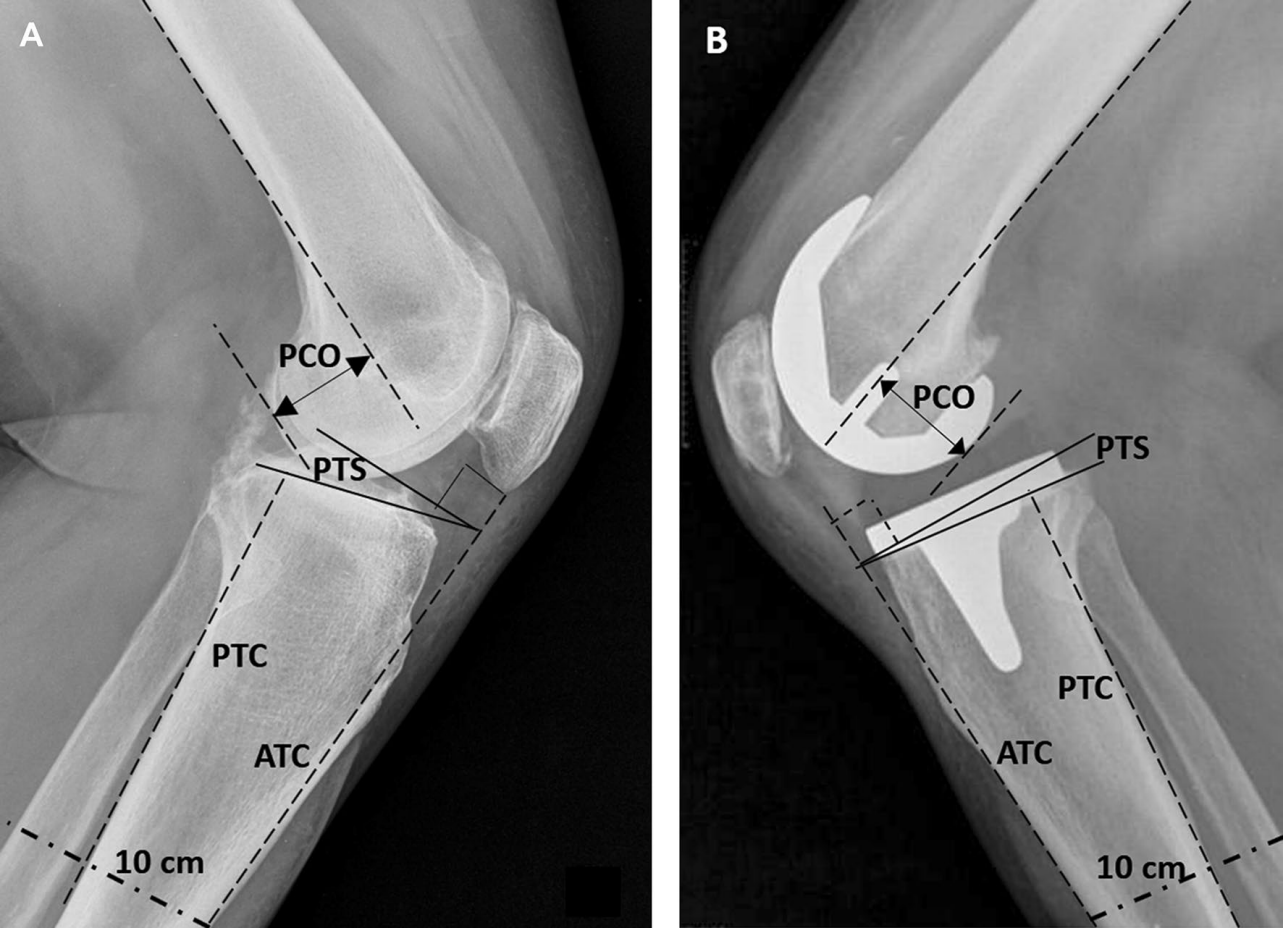
Preoperative (**A**) and postoperative (**B**) radiographs showing schematic measurements of posterior tibial slope (PTS) concerning the anterior and posterior tibial cortices (ATC, PTC), and femoral posterior condylar offset (PCO)

Radiological measurements were performed preoperatively and postoperatively by two independent surgeons. Digital radiographs were obtained according to the Digital Imaging and Communications in Medicine (DICOM) standards using a calibrated magnification. The measurements were taken in the computer using digital software (Centricity Universal Viewer Zero Footprint, GE Healthcare, Chicago, USA), with a measurement accuracy of 0.1 degrees and 0.1 mm. All measurements were performed on true lateral views of the knee (medial and lateral femoral condyles superimposed and the patellofemoral joint open and projected free). The PTS angle was calculated according to Utzschneider et al. [14] validated method on a short lateral radiograph for PTS angle measurement (Fig. 1). The anatomical reference lines were both the anterior and posterior tibial cortices at a 10 cm distance of the joint line, and the PTS value was the mean between the slopes with either reference line. The PTS was defined as the angle between a line perpendicular to the reference line and a line parallel to the tibial plateau or to the inferior border of the tibial tray. The ICC for PTS was 0.809.

The PCO was measured preoperatively as the distance between the tangent of the femoral diaphysis posterior cortex and the apex of the posterior femoral condyle preoperatively, or the apex of the posterior femoral component postoperatively [15]. The ICC for PCO was 0.812. The changes in PCO were negative if the final posterior condylar offset was decreased, and positive if increased.

### Statistical analysis

A priori sample size calculation was based on the primary outcome (KSS). A minimum of 135 patients was needed to detect the MCID and SBC in the KSS score for an 0.05 alpha error and 80% power. At least 148 patients were required, assuming a drop-out rate of 10%.

Statistical analyses were conducted with SPSS software v. 21.0 (SPSS Inc., Chicago, USA). A *p* value < 0.05 was considered significant in all analyses. The Kolmogorov–Smirnov test examined normal distribution. Comparisons between categorical variables were made with chi-square test, Fisher exact test or Mantel–Haenszel test, and *t* Student test or Mann–Whitney test was used for continuous variables. Comparisons between preoperative and last follow-up were made by paired *t* test or Wilcoxon signed-rank test. Correlations were assessed by Pearson or Spearman tests. A coefficient value < 0.5 was accepted as a weak or no correlation, 0.5–0.7 as moderate correlation, and > 0.7 as strong correlation. Multivariate analysis by logistic regression was used to analyze independent factors affecting final KSS scores. The final KSS was dichotomized as an improvement from preoperative to postoperative greater or less than SCB. Factors entered into the model were those with *p* < 0.10 in univariate analysis, and data were presented as odds ratio (OR) with a 95% confidence interval (CI).

## Results

One hundred and eighty-two patients meet the criteria. Of them, three died during the study period of causes unrelated to TKA, three were lost to follow-up, and two required TKA revisions (one early periprosthetic infection, and one periprosthetic femoral fracture). Additionally, 22 patients were also excluded by inadequate lateral radiographs. Thus, the study included 152 patients (98 females and 54 males) with a mean age of 74.6 (SD 7.2) years at the time of TKA. The mean postoperative follow-up was 5.3 (5–6) years.

Comparing patients who had an increase in PTS from preoperative to postoperative and those with a decrease, there were no significant differences in the preoperative characteristics (Table 1). At the final follow-up (Table 2), there were significant improvements from preoperative in KSS scores, ROM, and WOMAC-pain score (*p* = 0.001), exceeding the values of MCID and SBC in both groups. There were no significant differences between groups in the functional outcomes.

**Table 1:** Preoperative characteristics between patients with increased and decreased final PTS

Variables	Increased PTS (*n* = 88)	Decreased PTS (*n* = 64)	*p* value
Age (year)	75.3 (7.0)	73.9 (6.4)	0.209
Gender (F/M)	54/34	44/20	0.221
BMI (kg/m^2^)	29.0 (4.8)	28.2 (3.7)	0.248
KSS-knee	36.3 (10.9)	38.5 (13.7)	0.272
KSS-function	34.8 (10.3)	36.6 (10.1)	0.283
WOMAC-pain	40.4 (8.1)	42.5 (8.9)	0.132
WOMAC-function	39.8 (9.3)	41.4 (10.0)	0.317
ROM (º)	79.9 (9.8)	81.2 (10.1)	0.429
FT alignment (º)	Varus 5.9 (4.4)	Varus 6.5 (3.1)	0.325

*FT* femorotibial. Continuous data as mean (SD)

**Table 2:** Outcomes between patients with increased and decreased final PTS

Variables	Increased group	Decreased group	*p* value
KSS-knee	88.1 (7.6)	86.9 (8.0)	0.347
KSS-function	87.6 (8.1)	85.7 (9.0)	0.177
WOMAC-pain	88.1 (6.9)	86.2 (7.3)	0.103
WOMAC-function	86.5 (7.0)	84.7 (7.6)	0.134
ROM (º)	110.0 (10.1)	107.8 (10.7)	0.197
FT alignment (º)	Valgus 5.8 (2.6)	Valgus 6.1 (3.2)	0.538
PTS change (º)	3.9 (2.4)	4.9 (3.3)	0.041
PCO change (mm)	2.6 (1.1)	2.9 (1.8)	0.240

Continuous data as mean (SD)

Comparing patient groups according to the PTS change amount from preoperative to postoperative, there were no significant preoperative differences between groups (Table 3). At the final follow-up (Table 4), there was a significant improvement from preoperative in KSS scores, ROM, and WOMAC-pain score (*p* = 0.001), exceeding the values of MCID and SBC in either group. However, the final mean WOMAC-function score was significantly greater in the group of patients with PTS change ≤ 4º than in the group with PTS change > 4º (*p* = 0.024), although the mean difference was only 2.6 points.

**Table 3:** Preoperative characteristics of the groups according to the PTS change amount from preoperative to postoperative

Variables	Change ≤ 4º (*n* = 79)	Change > 4º (*n* = 73)	*p* value
Age (year)	72.8 (6.1)	74.5 (5.9)	0.083
Gender (F/M)	53/26	51/22	0.730
BMI (kg/m^2^)	28.7 (3.6)	29.8 (4.1)	0.081
KSS-knee	35.8 (11.2)	37.1 (14.9)	0.488
KSS-function	36.6 (9.5)	37.3 (9.7)	0.653
WOMAC-pain	39.7 (7.4)	41.1 (7.8)	0.258
WOMAC-function	40.9 (7.9)	42.2 (8.1)	0.826
ROM (º)	80.8 (9.0)	82.0 (9.5)	0.426
FT alignment (º)	Varus 6.3 (3.1)	Varus 6.0 (2.9)	0.538

*FT* femorotibial. Continuous data as mean (SD)

**Table 4:** Outcomes of the groups according to the PTS change amount from preoperative to postoperative

Variables	Change ≤ 4º (*n* = 79)	Change > 4º (*n* = 73)	*p* value
KSS-knee	87.3 (7.4)	86.6 (6.9)	0.547
KSS-function	88.2 (8.3)	86.6 (7.9)	0.225
WOMAC-pain	87.3 (6.4)	86.3 (6.9)	0.356
WOMAC-function	86.7 (7.1)	84.1 (7.0)	0.024
ROM (º)	109.0 (9.9)	108.4 (9.8)	0.708
FT alignment (º)	Valgus 5.9 (1.3)	Valgus 5.5 (1.4)	0.071
PTS change (º)	3.2 (1.3)	5.7 (1.8)	0.001
PCO change (mm)	2.3 (0.4)	2.5 (0.8)	0.057

Data as mean (SD)

Overall, the final mean PTS change from preoperative to postoperative was not significantly correlated with the final KSS (*r* = 0.23; *p* = 0.469), WOMAC (*r* = 0.71; *p* = 0.617) or ROM (*r* = 0.14; *p* = 0.169) in either group. A multivariate analysis was used to identify factors influencing an improvement from preoperative to postoperative greater than SCB (Table 5). No significant predictors were found, especially the PTS change (OR 1.08; 95% CI 0.70–1.40; *p* = 0.061).

**Table 5:** Multivariate analysis for final KSS greater than SCB

Factors	Univariate			Multivariate	
	≥ SCB (*n* = 116)	< SCB (*n* = 36)	*p* value	OR (95% CI)	*p* value
Age	71.6 (6.9)	74.0 (7.6)	0.027	0.12 (0.04–8.71)	0.724
BMI	28.3 (4.4)	30.1 (6.4)	0.058	1.08 (0.13–4.25)	0.691
Pre KSS-function	37.1 (7.9)	34.2 (9.8)	0.071	1.67 (0.05–4.39)	0.728
Pre WOMAC-function	43.3 (7.8)	40.6 (8.8)	0.080	1.91 (0.25–7.14)	0.642
Pre ROM	81.8 (9.6)	78.3 (10.4)	0.078	2.11 (0.95–7.14)	0.671
Pre PTS	7.1 (4.7)	8.3 (3.2)	0.085	0.93 (0.52–5.27)	0.167
PTS change	3.4 (2.0)	6.7 (2.7)	0.001	1.08 (0.70–1.40)	0.061

Continuous data as mean (SD)

*SCB* substantial clinical benefit, *Pre* preoperative, *OR (CI)* odds ratio (confidence interval)

Only factors with univariate *p* < 0.10 were included

## Discussion

The main finding in the present study was that the change in PTS from preoperative to postoperative did not significantly influence final functional outcomes. Although other studies [6, 16] found similar short-term results, to our knowledge, this is the first prospective study with a 5 year follow-up in analyzing the effect of the postoperative PTS change on the functional outcomes after CR-TKA.

Many studies have analyzed the influence of PTS following CR-TKA, but most were based on cadavers, biomechanical or computer models [9, 17, 18]. The PTS has been reported to be an important factor influencing the knee flexion after TKA [7, 19, 20], but the influence on other functional outcomes has been poorly analyzed. After a comprehensive review of the literature, only 2 prospective [2, 6] and 2 retrospective [3, 16] studies have clinically analyzed that influence. In prospective studies on CR-TKA, Malviya et al. [6] reported a moderate correlation between postoperative PTS and ROM, while Fujimoto et al. [2] found a high correlation. However, Malviya et al. [6] used a mix of fixed and mobile bearing TKA. In addition, these two studies had a follow-up of only 12 months, and other functional outcomes were not reported.

As in the present study, Seo et al. [16] found no significant difference in functional scores as measured with the KSS comparing changes in PTS higher or lower than 3º in a retrospective study of CR-TKA. In another study using a tibial cut parallel to the native anatomical slope, Howard et al. [3] reported that the patients who postoperatively had reproduction within 3º of their native PTS resulted in significantly better KSS, and WOMAC scores than those patients who had a PTS change of more than 3°. However, those differences were small and clinically irrelevant. Kansara et al. [4], in a small study of PS-TKA comparing cutting block at 0º and 5º PTS, found no significant difference in Hospital of Special Surgery (HSS) score after a 3 month follow-up.

A standardized PTS of 5º was used in the present study, similarly to other authors [2, 4, 7]. Lee et al. [5], in a retrospective study of PS-TKA with 2 year follow-up, reported that a PTS change of up to 7º did not affect postoperative ROM or functional outcomes measured with KSS and WOMAC scores. Conversely, some authors [17, 21] reported that an increase in PTS between 8 to 10º from native to postoperative had a significant impact on the anteroposterior kinematics of the CR-TKA in the mid-flexion range, but not in the high-flexion, while a decrease of 5º could reduce the flexion [22].

Some authors recommended increasing the PTS in CR-TKA to improve postoperative ROM [9, 10]. Nevertheless, an excessive increase in PTS could lead to a more significant flexion gap than the extension gap [23]. Other kinematic studies showed that incremented PTS resulted in a required lower force of the quadriceps to carry out the knee extension [2, 8] with a low chance of bearing impingement of the posterior femur [18]. Nevertheless, the benefit of increased PTS on the ROM was not demonstrated in other studies of CR-TKA [4, 6]. Conversely, other authors advised restoring the native PTS to optimize soft-tissue balance, and postoperative function [3, 8]. However, high variability in native anatomical tibial slope with 35% of people having a PTS less than 4° or more than 10° has been described [24, 25].

The present study has several limitations. Firstly, this was a non-randomized study. Lateral knee radiographs were used in this study to assess PTS, and the tibial rotation may influence that measurement. However, lateral radiographs with inadequate technique were excluded, and the ICC was checked to ensure the reliability of measurements. On the other hand, measurements were made on short knee radiographs but Utzschneider et al. [14] demonstrated a high correlation with the CT images. PTS measurements of both medial and lateral knee compartments were not performed, but several studies using radiographs and magnetic resonance images have reported no significant difference between medial and lateral PTS [14, 26]. Several methods for PTS measurement on radiographs have been described with reference lines in the anterior tibial cortex, posterior tibial cortex or longitudinal midline axis of the tibia [27]. High variability of measurements has been found between those methods [28]. In the present study, PTS was obtained according to a validated radiological method on short radiographs with a high correlation with CT [24], and provided a reliable level of accuracy [27]. Although CT imaging may have more excellent reliability [14], it is not routinely used to assess TKA in clinical practice because conventional radiographs have lower costs and radiation doses. Different prosthetic designs could provide also varying results in terms of functional outcomes or ROM, and conclusions of the present study could not be extrapolated to the posterior-stabilized designs. Nevertheless, further prospective comparative studies are needed to confirm the results.

### Conclusion

The PTS change between preoperative and postoperative has no influence on the functional outcomes using a CR-TKA. A standardized PTS regardless of the native is a reliable procedure for primary CR-TKA.
